# Personality Moderates Intra-Individual Variability in EEG Microstates and Spontaneous Thoughts

**DOI:** 10.1007/s10548-023-01019-x

**Published:** 2023-12-01

**Authors:** Miralena I. Tomescu, Claudiu Papasteri, Alexandra Sofonea, Alexandru I. Berceanu, Ioana Carcea

**Affiliations:** 1 Department of Psychology, Faculty of Educational Sciences, University “Stefan cel Mare” of Suceava, Suceava, Romania; 2 Departement of Research and Development, CINETic Center, National University of Theatre and Film “I.L. Caragiale”, Bucharest, Romania; 3 Department of Cognitive Sciences, Faculty of Psychology and Educational Sciences, University of Bucharest, Bucharest, Romania; 4 Department of Pharmacology, Physiology, and Neuroscience, Rutgers Brain Health Institute, New Jersey, NJ USA

**Keywords:** EEG microstates, Intra-individual variability, Personality traits, Spontaneous thoughts

## Abstract

Variability in brain activity that persists after accounting for overt behavioral and physiological states is often considered noise and controlled as a covariate in research. However, studying intra-individual variability in brain function can provide valuable insights into the dynamic nature of the brain. To explore this, we conducted a study on 43 participants analyzing the EEG microstate dynamics and self-reported spontaneous mental activity during five-minute resting-state recordings on two separate days with a twenty days average delay between recordings. Our results showed that the associations between EEG microstates and spontaneous cognition significantly changed from one day to another. Moreover, microstate changes were associated with changes in spontaneous cognition. Specifically, inter-day changes in *Verbal* thoughts about *Others* and future *Planning* were positively related to bottom-up sensory network-related microstate changes and negatively associated with top-down, attention, and salience network-related microstates. In addition, we find that personality traits are related to inter-day changes in microstates and spontaneous thoughts. Specifically, extraversion, neuroticism, agreeableness, and openness to experience moderated the relationship between inter-day changes in EEG microstates and spontaneous thoughts. Our study provides valuable information on the dynamic changes in the EEG microstate-spontaneous cognition organization, which could be essential for developing interventions and treatments for neuropsychiatric disorders.

**Supplementary Information:**

The online version contains supplementary material available at 10.1007/s10548-023-01019-x.

## Introduction

Brain networks have the adaptability and flexibility to respond to internal and environmental demands to support adaptation, maintain homeostasis, and promote mental health. Unfortunately, inter-day variations in brain functioning are understudied, usually considered noise, and controlled as a covariate. However, studying intra-individual and inter-day variability in brain functioning can provide valuable insights into the dynamic nature of the brain. For example, seasonally driven changes modulated by high daily variations in light exposure and difficulties in circadian rhythm adaptations increase the homeostasis load and risk for mood disorders (Zhang and Volkow 2023). Similarly, adaptation to challenging life events requires developing new coping strategies that might affect the inter-day variability in network dynamics and cognition.

Spontaneous thoughts vary in content and dynamics, affect, temporal and social orientation, mental modality, and association with physiological states (Andrews-Hanna et al. 2013; Killingsworth and Gilbert 2010). However, less is known about if and to what degree our spontaneous thoughts vary between days and if these patterns of change, or what we are addressing here as inter-day within-subject variability, are correlated with patterns of change of the underlying brain network dynamics. Stability and inter-day variations in recurrent thoughts about past and future events about ourselves and others are necessary for goal-directed activity, personal development, and adaptation to environmental challenges. For example, stability of recurrent thoughts about the past and future are necessary for goal-directed activity however, a certain degree of inter-day variability might be necessary for changing one’s goal when internal needs change or flexible adaptations to environmental challenges are necessary. Moreover, in contexts of negative mood, spontaneous thoughts that are biased toward adverse future outcomes, like, for example, during ruminations, one of the main cognitive symptoms of mood disorders, a high degree of inter-day stability as opposed to high enough inter-day variability might further reinforce their negative outcome. In other words, less inter-day variability in these thought patterns might further promote ruminative and negative mood states. On the contrary, increased inter-day variability might promote the reduction of negative bias and insightful positive-oriented outcome thoughts. For example, specific types of mind-wandering activity are the primary target of cognitive psychotherapy for depression (Chaieb et al. 2022; Kovács et al. 2020) and mindfulness-based approaches (van der Velden et al. 2015). With treatment, changes in daily variations of spontaneous thought might increase, in addition to the reduction of negative bias. As a first step in assessing how therapeutic interventions might modulate inter-day variability and if this would be a prognostic indicator, it would be essential to establish if there are significant variations in mind-wandering and the underlying temporal dynamics of brain networks when looking at spontaneous thought content and dynamics sampled from different days.

Measures like the Amsterdam Resting-State Questionnaire (ARSQ) enable self-report quantifications of the min-wandering experience that reflects the physiological state (sleepiness, somatic awareness, comfort) of the individual, the content (self, other, health-oriented) and dynamic of their spontaneous thoughts (discontinuity of mind) (Diaz et al. 2014). The different dimensions of the mind-wandering experience show high correlations over long periods; however, health-related thoughts, comfort, and sleepiness varied significantly between sessions (Diaz et al. 2014). Sleep quality might explain this variability, as many ASRQ facets show significant differences between insomnia patients and healthy participants, except for *Planning* and thoughts about *Others* (Palagini et al. 2016). Moreover, spontaneous thoughts vary as a function of personality traits. For example, more Self-Directedness, a measure of how well an individual can adapt to challenges, is related to fewer thoughts about *Self*, *Others*, or future *Planning* (Diaz et al. 2014). Harm-Avoidance traits, related to neuroticism and harm avoidance, are negatively related to *Comfort* thoughts (Diaz et al. 2014). These results suggest that having less thought content focused on *Self*, *Others,* or *Planning* and focusing more on *Comfort* might contribute to our general adaptability and mental health. Furthermore, we previously showed that personality traits predict changes in spontaneous thoughts after a social imitation intervention to reduce stress, which was negatively associated with *Self* related-thoughts (Tomescu et al. 2022). The changes in mind-wandering patterns, such as about *Self*, were negatively associated with neuroticism and positively related to extraversion (Tomescu et al. 2022). In addition, we found a negative association between reduced *Self*-related thoughts and spontaneous temporal dynamics of large-scale neural networks as measured by EEG microstates (Tomescu et al. 2022). Specifically, the decrease in *Self*-related mind-wandering activity was associated with increased C microstates (Tomescu et al. 2022), related to the default-mode network—considered the “self-experience” network (Bréchet et al. 2019; Custo et al. 2017; Tarailis et al. 2023).

EEG microstates are brief patterns of stable electrical activity lasting tens to hundreds of milliseconds. There is internal reliability of the microstate analysis as a function of number of electrodes, strategy of clustering and algorithm (Khanna et al. 2014), thus making the microstates a good method to study clinical biomarkers for susceptibility of neuropsychiatry disorders, and treatment response, brain development and inter-individual variability (Chivu et al. 2023; da Cruz et al. 2020; Koenig et al. 2002; Rieger et al. 2016; Tomescu et al. 2014; Tomescu et al. 2015, 2022). Microstates are related to intrinsic neuro-psychological traits; however microstate dynamics can also capture the state related specificity of the resting-state. For example, the dynamics change as a function of the content of mind wandering experience and during recollection of past memories. An increase in microstate B was observed (Bréchet et al. 2019). Moreover, we found in a recent meta-analysis that B microstates show atypically increased presence in mood and anxiety disorders, positively associated with comorbidity and severity of depressive symptomatology (Chivu et al. 2023). Moreover, microstates reflect the underlying dynamic processes of the brain related to self-paced cognitive functions such as attention, perception, and memory (Michel and Koenig 2018; Tarailis et al. 2023). In addition, microstates’ temporal presence is modulated by thoughts drifting from the cognitive task and external stimuli. Indeed, self-reported spontaneous thought experiences during mind-wandering have often been associated with fast dynamics of ongoing brain activity as characterized by EEG microstates (Pipinis et al. 2017; Tarailis et al. 2021; Tomescu et al. 2022; Zanesco et al. 2021). For example, thinking about the *Self* is related to decreased visual-related network B microstates (negative correlation) and increased attention network D microstates (positive correlation) (Tarailis et al. 2023). Furthermore, subjective experience of thoughts focused on *Self*-related *Somatic Awareness* and biological rhythms, such as breath and heart rate, have been negatively related to C and F/E microstates (Pipinis et al. 2017; Tarailis et al. 2021; Tomescu et al. 2022; Zanesco et al. 2021) with sources in the dorsal anterior cingulate cortex, superior frontal gyrus, middle frontal gyrus, and insula (Britz et al. 2010; Custo et al. 2017) and associated with salience resting state networks with a critical role in the integration of interoceptive information with emotional salience (Britz et al. 2010). Furthermore, posterior default-mode network default-mode network (DMN)-related C microstates have also been positively associated with *Comfort*, lack of conscious experience (*Discontinuity of Mind*), and *Planning* (Pipinis et al. 2017; Tarailis et al. 2021; Tomescu et al. 2022; Zanesco et al. 2021). While some of these results are contradictory (*Comfort* is anti-correlated with *Discontinuity of Mind*) (Diaz et al. 2014), other studies report a negative association between C microstates and *Comfort* (Tarailis et al. 2021). One possible explanation regarding this discrepancy might be inter- and intra-individual variability, and one crucial question to address would be to see if there is significant variability.

The main goal of this study was to assess if there is significant inter-day variability, meaning if differences from one day to another are significantly different from zero in terms of spontaneous thoughts as measured by ASRQ and microstates temporal dynamics without an a priory on what would be the source of this variability. Then, it would be essential to establish if the inter-day variability in mind-wandering is significantly associated with the inter-day variability in EEG microstates. Finally, we investigated if personality predicts inter-day variability in microstates and spontaneous thoughts. With this goal, we performed moderation analyses with personality traits as moderators of the inter-day associated variability between microstates and spontaneous thoughts recorded during a five-minute freely mind-wandering state with follow-up self-reports of their spontaneous mental activity measured by the ARSQ. By examining how spontaneous thoughts and EEG microstate dynamics fluctuate between two days and as a function of personality traits would be the first step towards identify patterns and mechanisms underlying inter-day variability and their functional significance.

## Methods

### Ethical Considerations

The National University for Theater and Film I.L Caragiale Bucharest Ethics Committee has approved all methods and experiments and followed the guidelines of the Declaration of Helsinki. All participants provided written informed consent for their participation.

### Participants and Data Collection

Participants were recruited through advertisements within the University of Bucharest, the University of Theater and Film, the funding project's website https://www.stadproject.eu/, and the CINETic’s Research Center website https://cinetic.arts.ro/en. Participants came on two different days in the lab. The first day (D1) participants filled in the NEO PI-R questionnaire. On both D1 and day 2 (D2), participants performed an eyes-closed 5 min resting-state as they were instructed to let their thoughts wander. After the EEG recording of the resting-state, participants filled in the Amsterdam Resting-State Questionnaire to assess their spontaneous thoughts during the resting-state (Diaz et al. 2014). There was, on average, a 20 days delay between D1 and D2 and all recordings were performed in the afternoon.

The data analyzed here is part of the data collected and reported by Tomescu et al. (2022), and more details about the dataset can be found there (Tomescu et al. 2022). The dataset thus included 43 participants (24 men, 19 women), mean age = 25.7 (age range: 20–42), s.d. = 5.1. (Women mean age = 25.57, s.d. = 5.3; Men mean age = 25.9, s.d. = 5.1). The sample group included in the moderation analyses with personality traits comprised 37 participants (18 men and 19 women). Six participants were excluded from these analyses due to missing NEO-PI-R data.

EEG data sampled online at 1 kHz with a Cz reference were acquired in a dimly room using a 128-channel ANT Neuro Waveguard System (https://www.ant-neuro.com/). Participants sat in a comfortable, upright position and were instructed to stay as calm as possible, to keep their eyes closed, and to relax for five minutes without falling asleep.

The Amsterdam Resting-State Questionnaire 2.0 (ARSQ) is a self-report questionnaire that quantifies mind-wandering experiences along ten scales: Discontinuity of Mind, Theory of Mind (Others), Self, Planning, Sleepiness, Comfort, Somatic Awareness, Health Concern, Visual Thought, Verbal Thought (Diaz et al. 2014). Participants had to respond to a total of 30 items (3 per each scale), using a 5-point Likert Scale from “Completely Disagree” to “Completely Agree.” The scores for each scale were then summed up and reported. For each scale, the maximum possible score is 15, and the minimum possible score is 0. The ARSQ enable self-report quantifications of the min-wandering experience that reflects the physiological state (sleepiness, somatic awareness, comfort) of the individual, the content (self, other, health-oriented), and the dynamic of their spontaneous thoughts (discontinuity) (Diaz et al. 2014)^.^The internal consistency was measured using Cronbach’s alpha, and the results show medium to high internal consistency, see Table S1. Previously Diaz et al. (2014) reported high correlation between the content and dynamic scale, while the scales reflecting more physiological states showed low correlation from data sampled on two different days (Diaz et al. 2014).

The Neo Personality Inventory–Revised (NEO PI-R) is a 240-item personality inventory with cross-culturally established properties and validity (McCrae et al. 2005) that assesses the Big Five Model domains of Neuroticism, Extraversion, Openness to Experience, Agreeableness, and Conscientiousness with the six factors for each domain. Participants responded on a 5-point Likert Scale ranging from 0 (strongly disagree) to 4 (strongly agree). The internal consistency was measured using Cronbach’s alpha, and the results show high internal consistency for each facet of the questionnaire; see Table S1 for more information.

### EEG Data Processing

The EEG datasets were band-pass filtered offline between 1 and 40 Hz with an additional notch at 50 Hz. EEG periods of movement contamination or other artifacts were marked and excluded from the analyses. To remove the oculomotor artifacts such as saccades and eye blinks, as well as the cardiac artifacts (ECG), we applied the Infomax-based Independent Component Analysis (ICA) (Jung et al. 2000). Bad or noisy electrodes were interpolated using a 3-D spherical spline (Perrin et al. 1989) and were recomputed to the common average reference. The data were then down-sampled to 125 Hz for further analysis.

The local maxima of the Global Field Power (GFP) show an optimal signal-to-noise ratio in the EEG (Murray et al. 2008). The EEG signal was extracted at the corresponding time frame of GFP peaks. Only the time points of GFP peaks were submitted to a modified k-means cluster analysis to identify the most representative classes of stable topographies (Murray et al. 2008).

The k-means clustering was performed in two steps: first, at the individual level, and second, at the group level by clustering all individual dominant topographies with varying clusters. To determine the optimal number of clusters at the individual and the group level, we used the criteria implemented in Cartool (a free academic software developed by Denis Brunet; cartoolcommunity.unige.ch), based on seven maximally independent criteria: Davies and Bouldin, Gamma, Silhouette, Dunn Robust, Point-Biserial, Krzanowski-Lai Index, and Cross-Validation (Custo et al. 2017).

In the first part of the microstate analysis, only GFP peaks were submitted to the k-means clustering. However, in the second part of the analysis, during the fitting process of the microstates, the entire EEG of participants was used, excluding only the marked artifact epochs. A temporal smoothing (window half-size 3-time frames (24 ms), Besag factor of 10, and a rejection of small time frames (when < 3, i.e., 24 ms) was applied. Subsequently, to quantify the temporal parameters of microstates, every time point of the individual data was assigned to the microstate cluster with which it correlated best (Brunet et al. 2011). A 0.7 correlation coefficient threshold was used to exclude transient periods of noise in the data. These periods were not labeled and were excluded from the analysis.

This fitting process enabled the determination of the duration and the occurrence of each microstate in each subject. The *mean duration* represents the average time (in ms) that a given microstate map was present without interruptions, i.e., the duration during which the subject remained in a specific state. The duration is one of the most commonly used parameters of the temporal structure of microstates and has repeatedly been shown to be associated with different vigilance conditions and symptoms of neuropsychiatric disorders (Khanna et al. 2014). The mean *occurrence* indicates the rate at which a given microstate occurs, i.e., how often the brain enters a specific state per second.

The free academic software Cartool (cartoolcommunity.unige.ch) was used for EEG data processing and microstate analysis (Brunet et al. 2011).

### Statistical Analyses

We tested data sets for Gaussian distribution using the Shapiro–Wilk normality test and concluded that our data have non-normal distribution. To assess the intra-individual variability between the two days, we compared microstate parameters and ARSQ spontaneous cognition between the two-time points Day 1 (D1) vs. Day 2 (D2). We utilized non-parametrical one-sample Wilcoxon tests; p-values were adjusted for multiple comparisons using false discovery rate (FDR) correction (*p* < 0.05). Statistical power was estimated by computing post-hoc testing and for N = 43 for the one-sample t-test change difference a Power (1-β error probability) = 0.93. For the moderation, we computed interaction for linear regression model for N = 37 and found a Power (1-β error probability) of 0.69 for a medium effect size.

To identify robust patterns of correlations between personality traits, spontaneous cognition, and the temporal dynamics of the EEG microstates, we used a multivariate approach called partial least squares (PLS) (Krishnan et al. 2011). PLS is a multivariate data-driven statistical technique that maximizes the covariance between two matrices by deriving *latent variables* (LVs), which are optimal linear combinations of the original matrices (Krishnan et al. 2011). PLS is a powerful technique for relating two sets of data (e.g., neuroimaging and behavioral data), even if these data show autocorrelation or multicollinearity (McIntosh and Misic 2013).

Each LV is characterized by a distinct EEG microstate pattern (EEG *loadings*) and a distinct behavioral profile (behavioral *loadings*). By linearly projecting each participant’s EEG and behavioral measures onto their respective loadings, we obtained individual-specific EEG microstates and behavioral *composite scores* for each LV. PLS seeks to find loadings that maximize across-participant covariance between the EEG microstates parameters and behavioral composite scores. The number of significant LVs was determined by a permutation test (1000 permutations). The p-values (from the permutation) for the first five LVs were corrected for multiple comparisons using a false discovery rate (FDR) of *q* < 0.05. To interpret the LVs, we computed Pearson's correlations between the original EEG data and EEG composite scores and between the original behavioral measures and behavioral composite scores for each LV. A large positive (or negative) correlation for a particular behavioral measure for a given LV indicates greater importance of the behavioral measure for the LV.

Similarly, a large positive (or negative) correlation for a particular EEG microstate parameter for a given LV indicates greater importance of the EEG microstate parameter for the LV. We used a bootstrapping procedure to estimate confidence intervals for these correlations, which generated 500 samples from subjects’ data. First, z-scores were calculated by dividing each correlation coefficient by its bootstrap-estimated standard deviation. Then, the z-scores were converted to p-values and FDR-corrected (*p* < 0.05) (Kebets et al. 2019).

We performed explorative moderation analysis to investigate if personality predicts the inter-day variability between the EEG microstates and spontaneous cognition. In the moderation models we used spontaneous cognition and EEG microstates as both dependent and independent variables in interaction with personality facets of the NEOPI-R questionnaire.

## Results

**Fig. 1:**
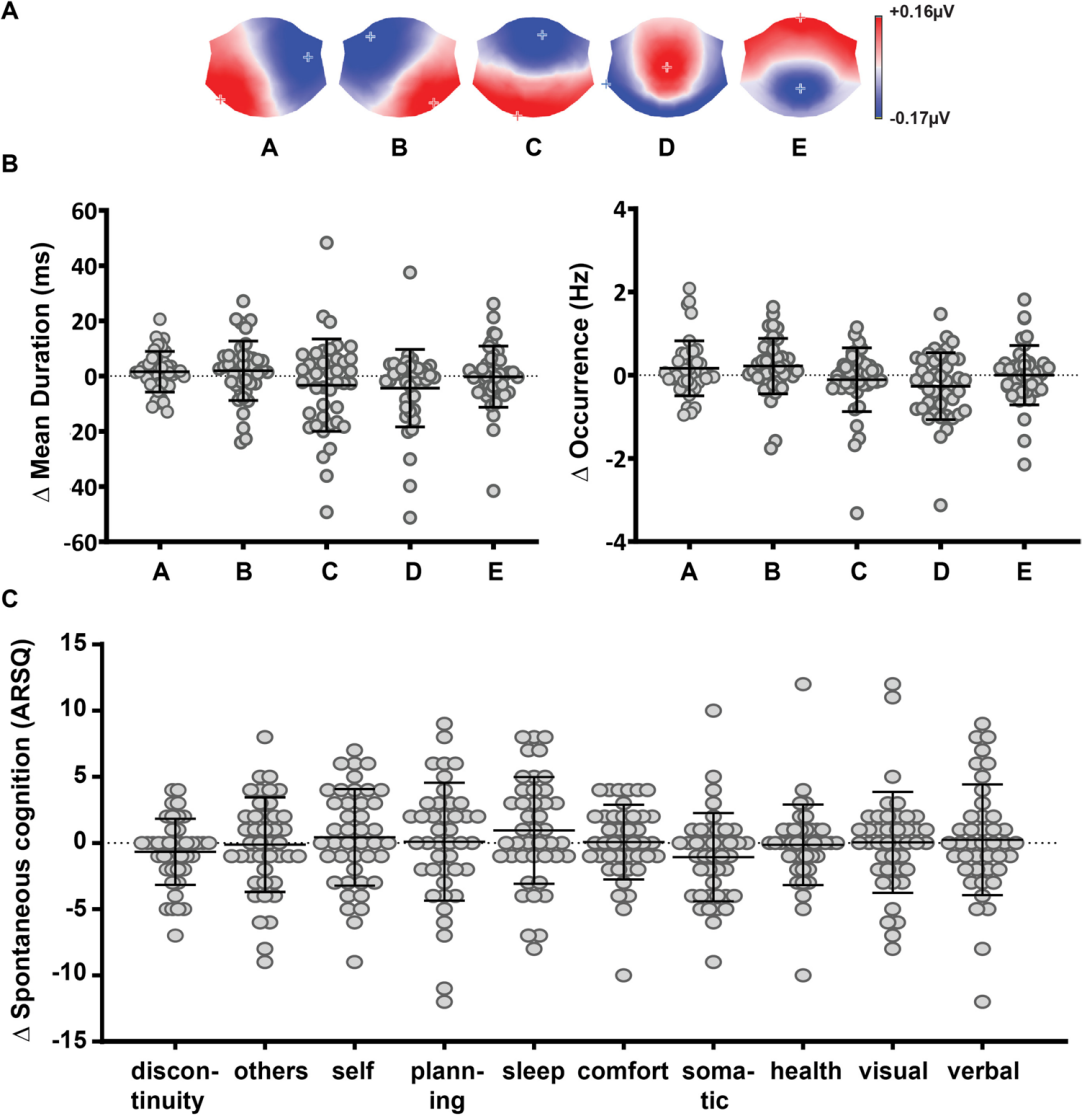
Intra-individual variability between 2 days in microstate dynamics and spontaneous cognition. **A** Dominant classes of microstates. **B** Microstate temporal dynamics D2–D1 difference scores. **C** Spontaneous cognition D2–D1 difference scores. Error bars are standard deviations

The EEG microstates in our dataset were identified using a set of seven independent criteria (detailed in Methods). First, we determined that five microstates can optimally describe group topographical EEG variability. Then, across both days and individuals (*N* = 43), the cluster analysis robustly identified A, B, C, D, and E prototypical microstates that explained 81.9% of the variance (Fig. 1A). We then analyzed inter-day changes in the *duration* and *occurrence* of these EEG microstates by calculating change scores (D2–D1) (Fig. 1B**)**. We find that absolute values of D2–D1 scores for both *mean duration* and *occurrence* of all microstates are significantly larger than zero (*mean duration* |D2–D1| scores for microstate: A = 5.61 ± 4.7 ms, p < 0.0001, one sample Wilcoxon test; B = 8.0 ± 7.16 ms, p < 0.0001; C = 12.02 ± 11.69 ms, p < 0.0001; D = 8.69 ± 11.57 ms, p < 0.0001; E = 7.31 ± 8.08 ms, p < 0.0001; *occurrence* |D2–D1| scores for microstate: A = 0.44 ± 0.5 Hz, p < 0.0001; B = 0.49 ± 0.48 Hz, p < 0.0001; C = 0.46 ± 0.6 Hz, p < 0.0001; D = 0.61 ± 0.55 Hz, p < 0.0001; E = 0.48 ± 0.5 Hz, p < 0.0001). These results indicate significant intra-individual variability of resting-state EEG microstate dynamics. Across subjects, however, microstate changes are bidirectional (either D2 > D1 or D2 < D1), and the coefficients of variation are high (*mean duration* CV for D2–D1 scores for microstate: A = 4.66, B = 5.77, C = 5.44, D = 3.29, E = 40.43; *occurrence* CV for D2–D1 scores for microstate: A = 3.91, B = 3.29, C = 7.16, D = 3.10, E = 193.36). Therefore, we can conclude that there is both intra- and inter-individual variability in EEG microstate dynamics.

The observed variability in neural data could reflect inter-day differences in spontaneous thoughts (Fig. 1C**)**. Thus, we looked at inter-day changes (D2–D1) in self-reported spontaneous thoughts. The results show that absolute values of D2–D1 scores are significantly different than zero (*Discontinuity* = 1.93 ± 2.0, p < 0.0001, *Others* = 2.72 ± 2.23, p < 0.0001, *Self* = 3.02 ± 2.3, p < 0.0001, P*lanning* = 3.32 ± 2.87, p < 0.0001, S*leep* = 3.16 ± 2.5, p < 0.0001, *Comfort* = 2.04 ± 1.8, p < 0.0001, *Somatic Awareness* = 2.48 ± 2.3, p < 0.0001, *Health* = 1.74 ± 2.4,p < 0.0001, *Visual* = 2.58 ± 2.8, p < 0.0001, *Verbal* = 3.04 ± 2.9, p < 0.0001). Moreover, the coefficients of variation are high (D*iscontinuity* = 3.32, *Others* = 21.77, S*elf* = 16.46, *Planning* = 27.17, S*leep* = 4.53, *Comfort* = 60.07, *Somatic Awareness* = 3.01, *Health* = 25.88, *Visual* = 55.1, V*erbal* = 61.32). We observe high intra- and inter-individual variability in spontaneous thoughts.

### The Relationship Between Spontaneous Cognition and EEG Microstate Varies Between D1 and D2

We performed two separate PLS analyses for each time point to fully understand intra-individual variability in the relationship between spontaneous cognition and microstate dynamics. On the first day of recording (D1), there was a significant PLS relation between spontaneous cognition as measured by the ARSQ and EEG microstate parameters (LV1-p = 0.003, FDR corrected, explaining 45% of the covariance). Figure S1A–D illustrates the significant association between EEG microstates D and spontaneous thoughts about *Self, Somatic Awareness, Planning,* and *Comfort*. On the second day (D2), we found a significant PLS association (LV1-p = 0.00, FDR corrected, explaining 56% of the covariance) between microstates and spontaneous thoughts about *Comfort, Discontinuity of Mind*, and *Visual* and *Verbal* thoughts for more details, see Fig. S1E–H.

**Fig. 2:**
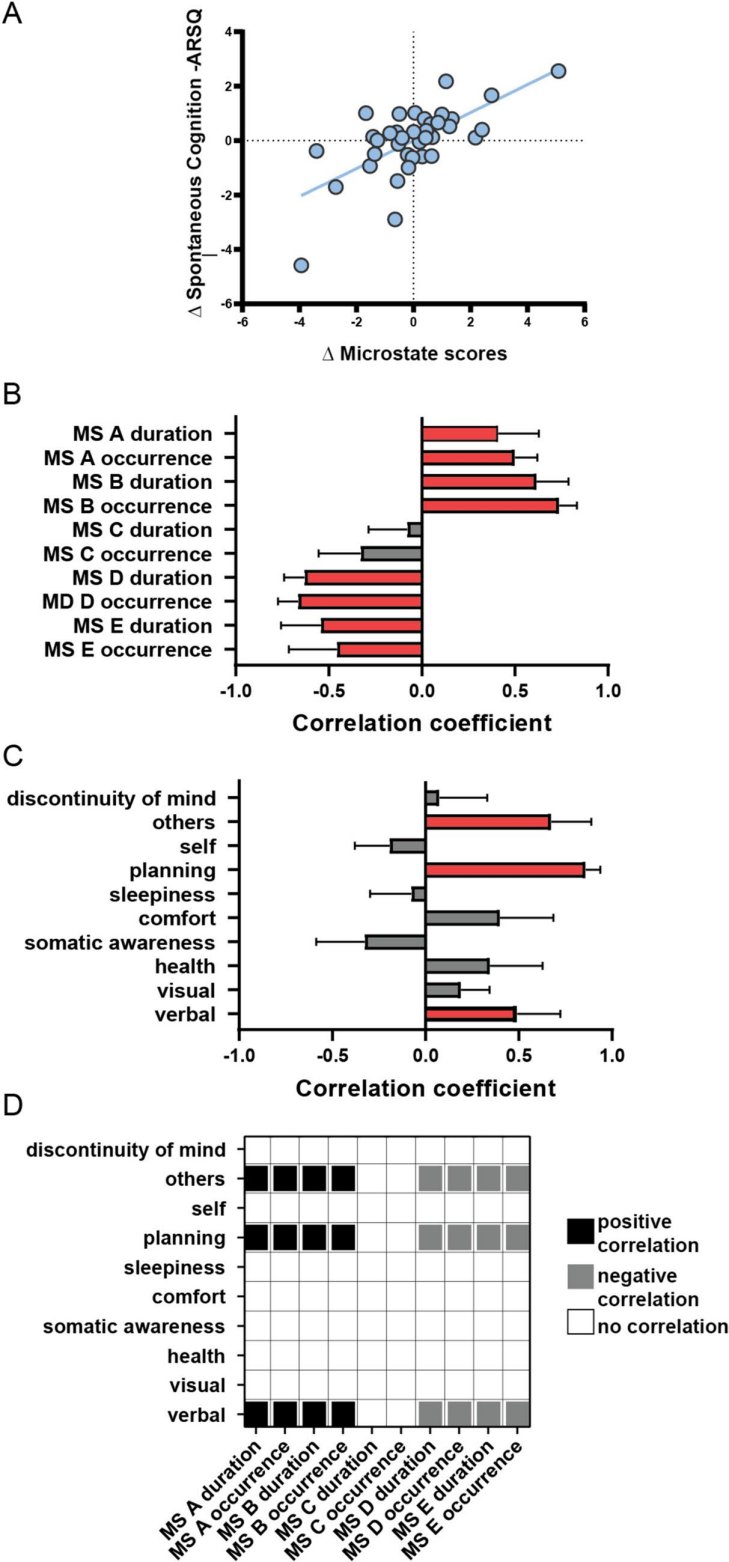
Association between inter-day changes (D2–D1) in spontaneous cognition and microstates dynamics. **A** D2–D1 correlation between individual-specific ARSQ scores and microstate parameters. **B** D2–D1 correlations between original and composite microstate duration and occurrence scores. Red color bars represent significant correlation coefficients **C** D2–D1 correlations between original and composite ARSQ scores. Errors represent SD. **D** D2–D1 specific associations between microstate dynamics and spontaneous thoughts

Given the different patterns of associations between D1 and D2, we investigated whether microstates and spontaneous cognition varied together. We performed an additional PLS analysis to assess the association between D2–D1 change scores in microstates and spontaneous cognition. The results show that some of these changes are related. For example, daily changes in A and B microstates are positively associated with change scores in thoughts about the future (*Planning*), about other people (*Others*), and verbal thoughts (*Verbal*). In contrast, the variability of D and E microstates is negatively associated with these spontaneous thoughts. The results of the significant PLS (p = 0.01, FDR corrected, 64% explained variance) are shown in Fig. 2.

### Fantasy and Trust Moderate Associations Between Microstates and Spontaneous Thoughts

**Fig. 3:**
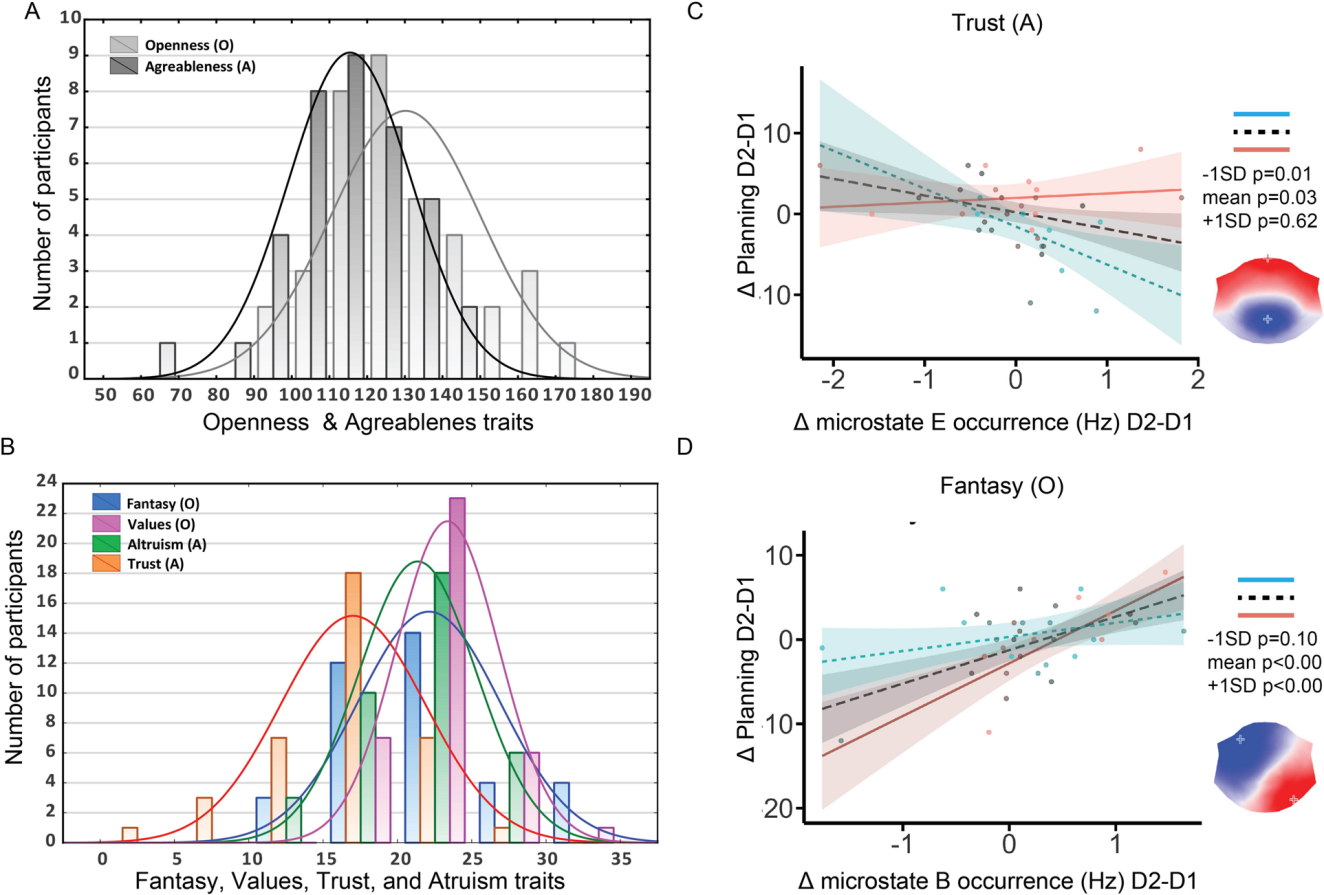
Personality moderates inter-day associated changes in spontaneous thoughts and microstate occurrence. **A** Histogram of Openness to Experiences and Agreeableness personality traits. **B** Histogram of Openness to Experiences facets Fantasy and Values, and Agreeableness facets Altruism and Trust personality traits. **C** Trust personality traits moderate inter-day changes in Planning and microstate E occurrence. **D** Fantasy personality traits moderate the association between inter-day changes in Planning and microstate B occurrence. Blue dots and regression lines represent individuals with one standard deviation below the mean (−1SD) on personality trait distribution. Red dots and regression lines represent individuals with one standard deviation above the mean (+ 1SD) on personality traits

We performed moderation analyses to see if personality predicts the significant PLS (Fig. 3) D2–D1 associations between microstates (A, B, D, and E) and *Planning*, *Others*, and *Verbal* spontaneous thoughts.

Personality traits moderate the relationship between microstates B, and E and spontaneous thoughts about future (*Planning*) D2-D1 scores (Fig. 3, Table 1, Fig. S2, Table S5).

**Table 1:** Moderation analysis results for B, and E microstates & *Planning*

*Slope*	*Predictors*	*Estimates*	*CI*		*t*	*p*
Planning	Fantasy × MS B occurrence	0.48	0.15	0.81	2.98	**0.005**
−1SD	Low fantasy	1.68	− 0.37	3.73	1.67	0.10
Mean	Mean fantasy	3.97	2.04	5.89	4.19	**0.00**
+ 1SD	High fantasy	6.26	3.41	9.10	4.47	**0.00**
Planning	Trust × MS E occurrence	0.54	0.08	1.00	2.37	**0.024**
−1SD	Low trust	− 4.69	− 8.19	− 1.19	− 2.73	**0.01**
Mean	Mean trust	− 2.07	− 3.97	− 0.17	− 2.22	0.03
+ 1SD	High trust	0.55	− 1.71	2.81	0.49	0.62

Text in bold highlights significant *p*-values

More specifically, we found a more significant negative association between *Planning* and microstate E in low Agreeableness Trust trait (−1SD) individuals. We also observed that high Fantasy (+ 1SD) individuals showed more positive significant associations between *Planning* and microstates B (Fig. 3C, D** , **Table 1). These results remained significant even after outlier elimination. In addition, we found that individuals with low Openness traits (−1SD) and Values (−1SD) showed more significant associations between *Planning* and microstates A. Low Openness and low Agreeableness, such as Values (−1SD), Trust (−1SD) and Altruism (−1SD) traits individuals showed more positive significant associations between *Planning* and microstates B (Fig. S2). However, these results did not survived the outlier correction analysis.

### Vulnerability to Stress, and Gregariousness Moderate Associations Between Microstates and Spontaneous Thoughts

We performed moderation analyses to see if personality moderates the changes in microstates and spontaneous thoughts that do not appear to be associated in the PLS analysis (Fig. 2). For example, as a function of personality traits, association patterns might occur in opposite directions (negative vs. positive associations), cancel each other and explain why we did not find significant associations between D2–D1 changes in microstates, and *Discontinuity of Mind, Self, Sleepiness, Comfort, Health, Somatic Awareness,* and *Visual* spontaneous thoughts.

**Fig. 4:**
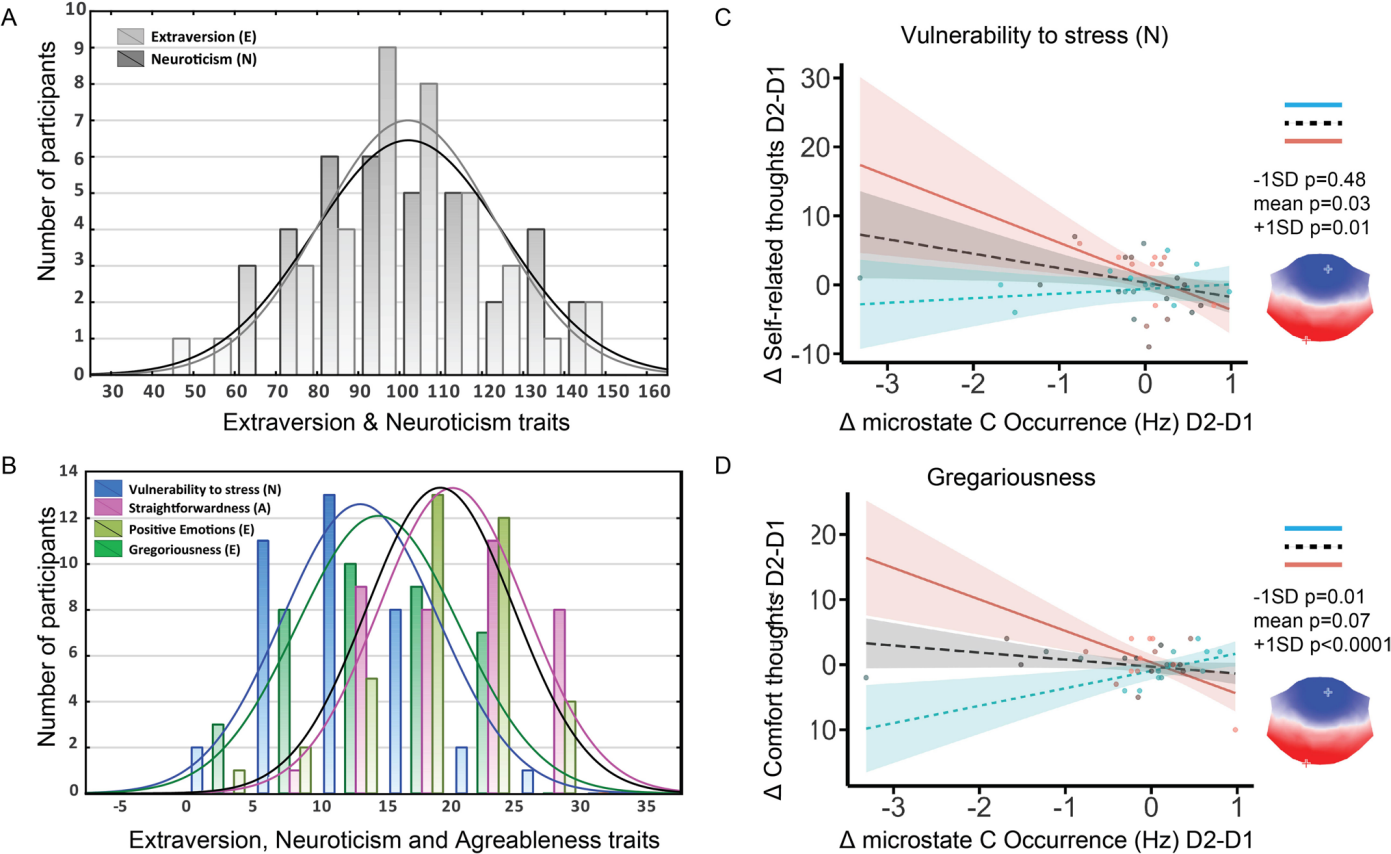
Personality moderates inter-day associated changes in spontaneous thoughts and microstate occurrence. **A** Histogram of Extraversion and Neuroticism personality traits. **B** Histogram of Vulnerability to Stress, Straightforwardness, Positive Emotions, Gregariousness personality traits. **C** Vulnerability to stress personality traits moderates the association between inter-day changes in Self-related thoughts and microstate C occurrence. **D** Gregariousness personality traits moderate the association between inter-day changes in thoughts about Comfort and microstate C occurrence. Blue dots and regression lines represent individuals with one standard deviation below the mean (−1SD) on personality trait distribution. Red dots and regression lines represent individuals with one standard deviation above the mean (+ 1SD) on personality traits

Personality traits moderate the relationship between microstates C and D and spontaneous thoughts about *Self* and *Comfort* (Fig. 4**, **Table 2, Fig. S3, Table S6).

**Table 2:** Moderation analysis results for C microstate & *Self* and *Comfort*

*Slope*	*Predictors*	*Estimates*	*CI*		*t*	*p*
Self	Vulnerability to stress × MS C occurrence	− 0.47	− 0.85	− 0.1	− 2.55	**0.016**
−1SD	Low VulStress	0.67	− 1.24	2.58	0.71	0.48
Mean	Mean VulStress	− 2.1	− 3.99	− 0.2	− 2.25	**0.03**
+ 1SD	High VulStress	− 4.86	− 8.51	− 1.22	− 2.71	**0.01**
Comfort	Gregariousness × MS C occurrence	− 0.62	− 0.93	− 0.3	− 3.94	** < 0.001**
−1SD	Low Greg	2.67	0.81	4.53	2.92	**0.01**
Mean	Mean Greg	− 1.08	− 2.27	0.11	− 1.84	**0.07**
+ 1SD	High Greg	− 4.83	− 7.45	− 2.21	− 3.75	** < 0.001**

Text in bold highlights significant *p*-values

More specifically, we observed that individuals with high Vulnerability to Stress (+ 1SD) showed significant change scores associations between *Self* and microstates C (Fig. S3). Moreover, a significant reversed patterns between high (+ 1SD) and low (−1SD) Gregariousness traits of significant associations were found between *Comfort* and microstate C. These results remained significant after outlier elimination. More importantly, these association patterns explain why we did not find any significant association between thoughts about Comfort and microstates in the PLS association.

In addition, low Extraversion (−1SD), low Positive Emotions, and low Straightforwardness showed significant change scores associations between *Self* and microstates C and D (Figure S3). However, the opposite patterns of associations between *Self-*related scores and D were observed for low Vulnerability to Stress (−1SD), high extraversion (1 + SD), and high Positive Emotions (1 + SD). However, these results did not survive after outlier elimination.

## Discussion

Brain-mind inter-day variability might be essential to flexibly adapt to internal and environmental demands that support mental health. As a first step, in a sample of healthy individuals, we found significant variations in intra-individual self-reports of spontaneous cognition during resting state and temporal dynamics of EEG microstates from D1 to D2 (Fig. 1). Moreover, for each sample of the two days, a different pattern of association characterized the relation between spontaneous thoughts and the underlying dynamics of microstate activity (Figure S1). Investigating these specific changes, we found that inter-day changes in *Verbal* thoughts about *Others* and future *Planning* were positively associated with inter-day changes in A and B microstates (Fig. 2). The opposite was true for D and E microstates. Inter-day changes in D and E were negatively correlated with *Verbal, Others,* and *Planning* spontaneous thoughts.

Regarding their functional relevance, microstates A exhibited multisensory associations, including verbal, auditory, and phonological, while microstates B exhibited links to visual processing specifically associated with self-retrieval of autobiographical memories (Tarailis et al. 2023). The positive inter-day associations align with the observed functional relevance and further extend the notion that these microstates also play a role in imagining the future and planning accordingly. Moreover, these results sustain our hypothesis that inter-day variability might be essential for successful goal-directed activity and adaptation to environmental challenges. However, future research should address how the observed patterns of inter-day variability are associated with positive or negative outcomes in adaptive situations. Furthermore, the inter-day variability of microstate and spontaneous thoughts should be investigated in clinical studies to further disentangle the interaction with mood and anxiety disorders, as well as negative symptoms in schizophrenia as many clinical studies reveal significant modulations of B microstates (Chivu et al. 2023; Rieger et al. 2016).

The functional relevance of microstates D and E relates to sustained attention, executive functioning, and interoceptive and emotional information processing (Tarailis et al. 2023). Variations of these microstates were negatively associated with *Verbal, Others,* and *Planning* spontaneous thoughts. These results suggest that there might be less engagement of salience, attention, and executive functioning states during spontaneously imagining situations involving future social interactions, for example. Alternatively, these situations involve a low cognitive load that might vary as a function of the context. Indeed, both of these networks are task-positive, meaning that they tend to increase in presence while actively performing tasks; however, microstate E might also be involved in the processing of emotional information and was previously found to be positively associated with Comfort (Tarailis et al. 2023), it would be essential to assess inter-day variability by also taking into account modulations of emotional contexts such as during prolonged states of uncomfortable negative mood ruminative states. It has been suggested that atypically shortened E microstates might reflect a failure to map relevant bottom-up stimuli, resulting in hypervigilance in patients suffering from anxiety-related disorders like PTSD (Terpou et al. 2022). Microstate D decreased presence was also reported in patients suffering from major depression during both full-blown and symptom remission states, thought to reflect a continuous depression trait (Murphy et al. 2020). Thus, it is essential to investigate whether certain personality traits drive the observed variability.

We performed moderation analyses to explore whether personality might play a role in the inter-day D2–D1 associations between microstates and spontaneous cognition. The results showed that personality traits might predict the relationship between EEG microstates and spontaneous thought’s inter-day variability. More specifically, the inter-day variability positive association between bottom-up B microstates and thoughts about *Planning* (future, problem-solving, and things I need to do) was driven mainly by high day-dreamers (high Fantasy) (Fig. 3). These results are in line with previous work showing microstate B association with auto-biographical memory retrieval during resting-state further suggesting possible common underlying brain mechanism for remembering and imagining the future (Bréchet et al. 2019; Smallwood and Andrews-Hanna 2013; Tarailis et al. 2023). In addition, low Trust individuals mainly drove the inter-day variability negative relation between top-down salience network-related E microstates and thoughts about *Planning*. Given that *Planning* was previously negatively correlated with more Self-Directedness, a measure of how well an individual can adapt to challenges (Diaz et al. 2014), and E was suggested as a marker for anxiety (Chivu et al. 2023), it would be important for future studies to investigate how the inter-day variability in microstates and spontaneous thoughts modulated by personality is predicting behavior during challenging experiences.

Thoughts about *Self*, were also negatively associated with Self-Directedness. Here, we found that individuals most vulnerable to stress (high Vulnerability to Stress) show a negative relation between *Self*-thoughts and DMN-related C microstates. We previously showed that *Self*-related thoughts are significantly negatively associated with C-DMN microstates after a social imitation task (Tomescu et al. 2022). Moreover, we observed that more *Self*-related thoughts are associated with higher self-reported stress levels (Tomescu et al. 2022). Variability between C-DMN and *Self*-related thoughts might be an important treatment target and marker for preventing stress-related mental health disorders. Thus, future studies might focus on the content of self-related thoughts. In addition, investigating the relationship between C-DMN microstates and self-related thoughts in clinical populations would be highly important. Individuals at high-risk and schizophrenia patients robustly show increased C microstate dynamics (da Cruz et al. 2020; Rieger et al. 2016; M. Tomescu et al. 2014; Tomescu et al. 2015).

Finally, the level of gregariousness (extraversion) and the drive for human connection moderated the relationship between thoughts about *Comfort* and DMN-related C microstates. Less drive for social interaction (gregariousness) predicts a positive DMN-related C microstates to *Comfort* thoughts relation and the opposite was true for high gregarious individuals. As Harm-Avoidance traits are also negatively associated to *Comfort* thoughts (Diaz et al. 2014), more studies on modulating thoughts about *Comfort* and microstate C dynamics might contribute to understanding neuro-psychological mechanisms of general adaptability, mental health stability, and resilience.

## Limitations

Some limitations of this study should be acknowledged, like the relatively small size (N = 43) included. More studies are needed to confirm these results in a larger sample size. Moreover, long-term inter-day variability is needed to confirm these findings and overcome another limitation—only two samples were investigated here (D1 and D2). Future studies would also extend these findings by taking more samples with larger time delays between recordings. Further investigating the qualitative experience of self-related thought engagement in addition to the ARSQ questionnaire might reveal more information about the quality and quantity of inter-day spontaneous thought variability.

## Conclusions

In conclusion, the results of this study provide valuable information about the intra-individual variability and dynamic changes in the EEG microstate-spontaneous cognition organization, which could lead to the development of new interventions and monitoring applications for neurologic and psychiatric disorders.

### Supplementary Information

Below is the link to the electronic supplementary material.

Supplementary file1 (JPG 2056 KB)

Supplementary file2 (JPG 828 KB)

Supplementary file3 (JPG 893 KB)

Supplementary file4 (DOCX 598 KB)
