# Supplementary figures and images for: Personality Moderates Intra-Individual Variability in EEG Microstates and Spontaneous Thoughts
Source: Brain Topogr. 2023 Dec 1;37(4):524–35. doi: 10.1007/s10548-023-01019-x (PMC11199214; doi:10.1007/s10548-023-01019-x)

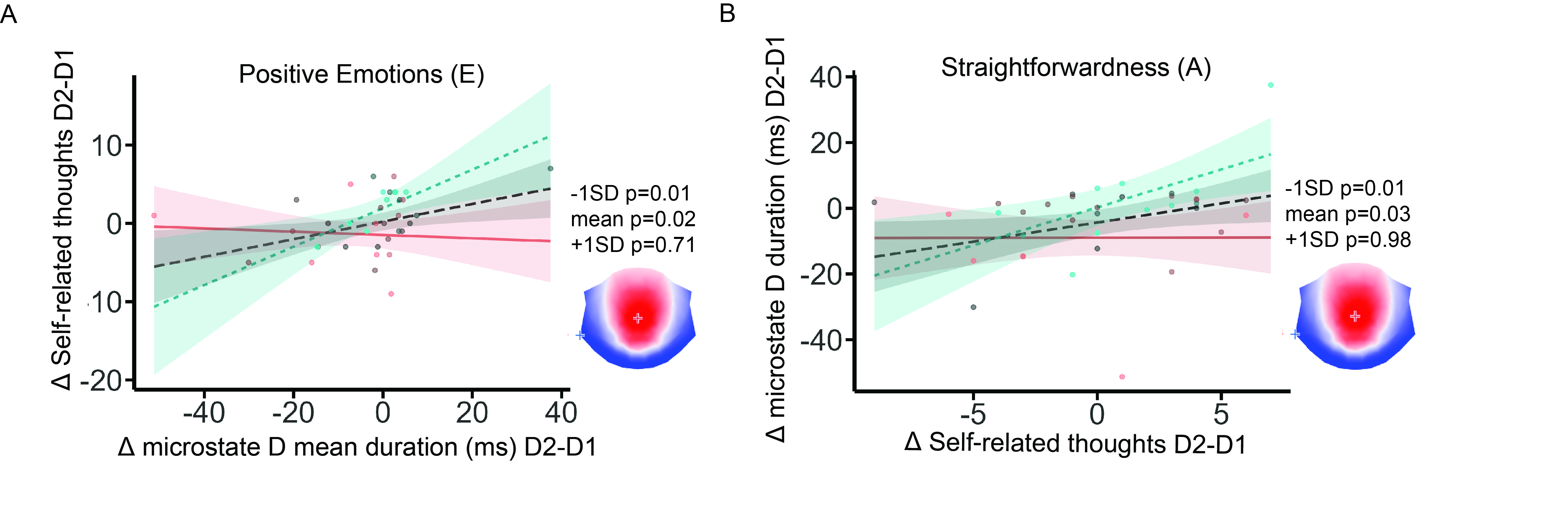

Supplement: Supplementary file 1 — Supplementary file1 (JPG 2056 KB) [file 10548_2023_1019_MOESM1_ESM.jpg]

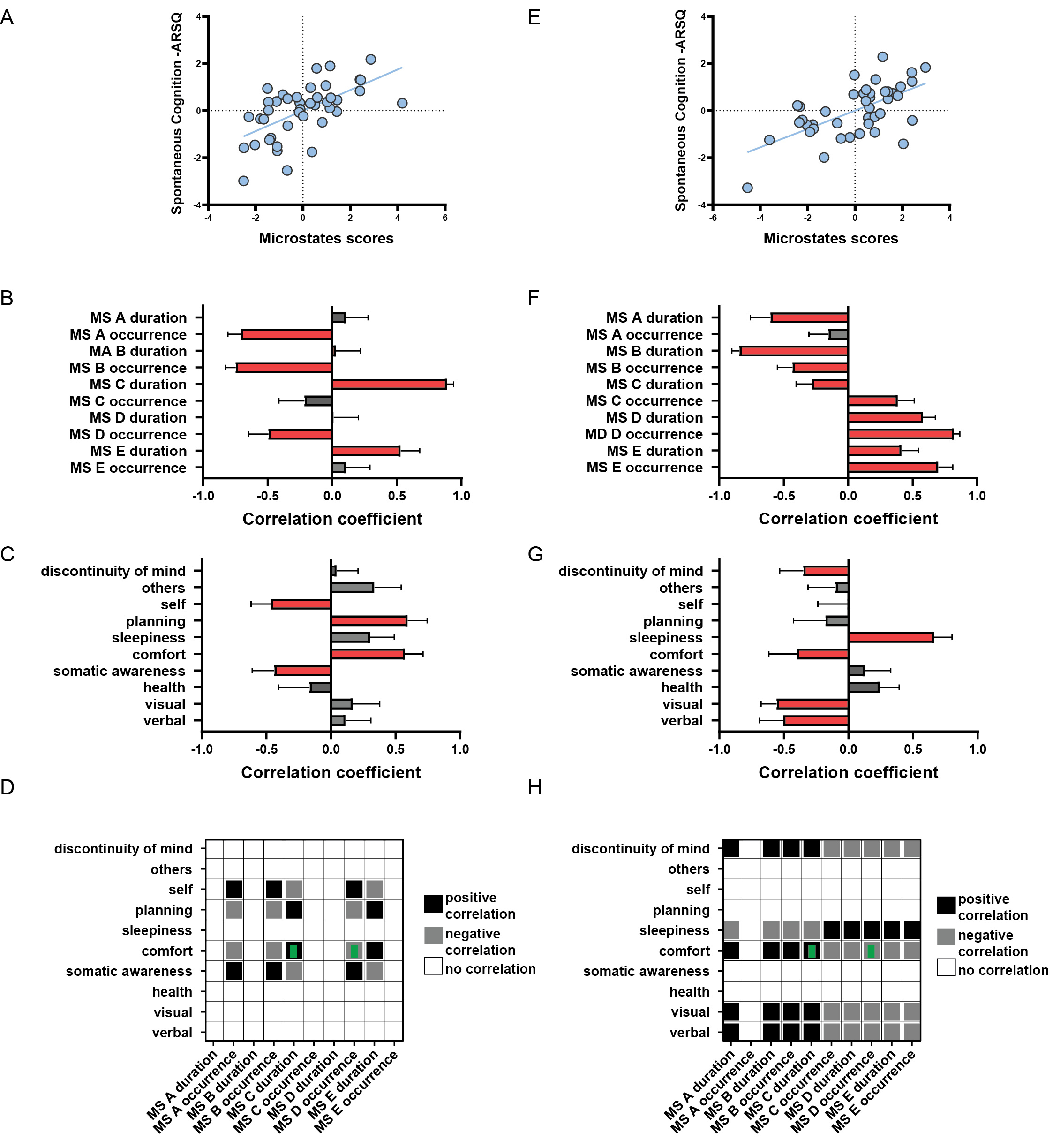

Supplement: Supplementary file 2 — Supplementary file2 (JPG 828 KB) [file 10548_2023_1019_MOESM2_ESM.jpg]

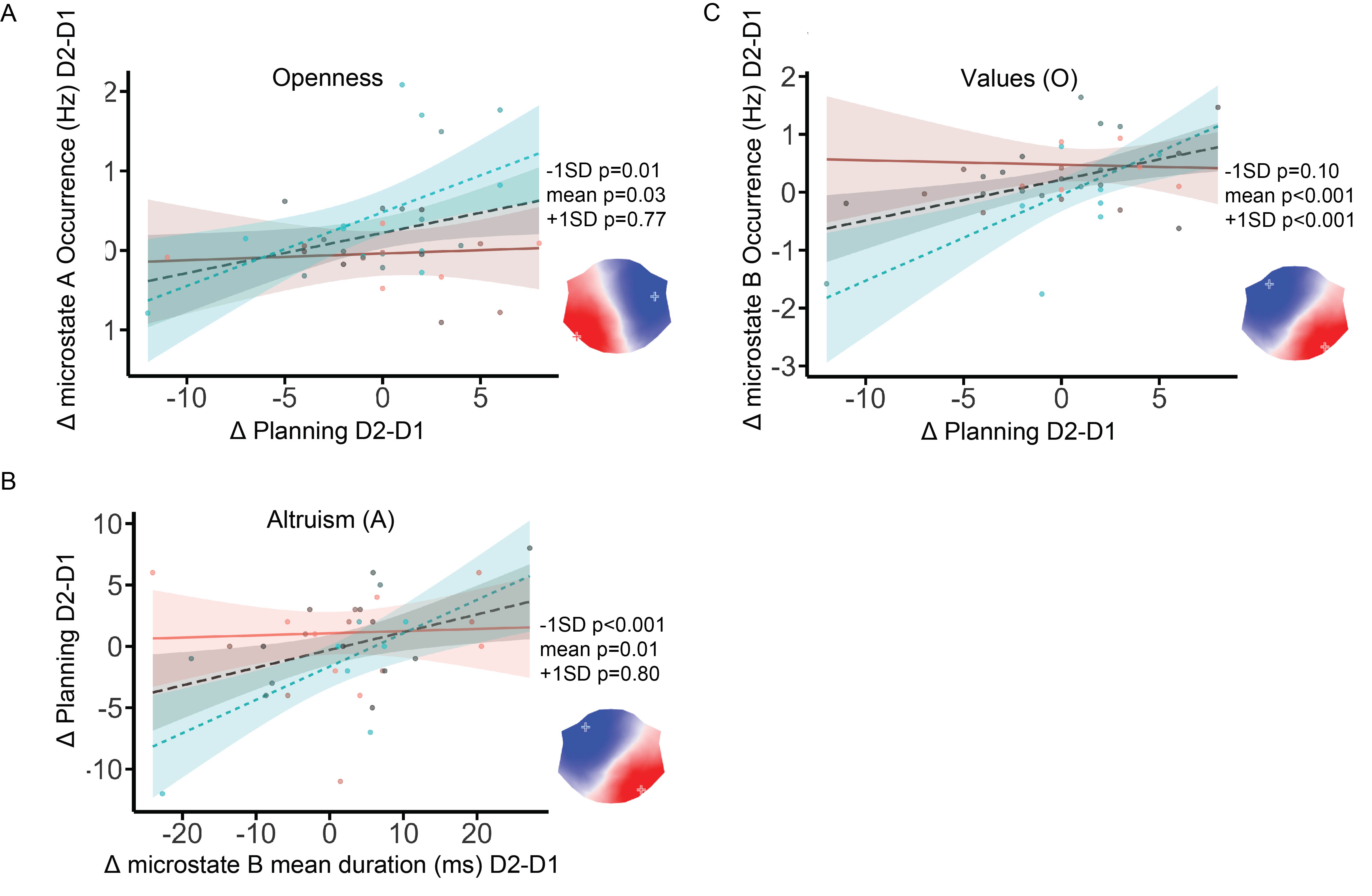

Supplement: Supplementary file 3 — Supplementary file3 (JPG 893 KB) [file 10548_2023_1019_MOESM3_ESM.jpg]
