# Supplementary material for: Personality Moderates Intra-Individual Variability in EEG Microstates and Spontaneous Thoughts
Source: Brain Topogr. 2023 Dec 1;37(4):524–35. doi: 10.1007/s10548-023-01019-x (PMC11199214; doi:10.1007/s10548-023-01019-x)
Supplement: Supplementary file 4 — Supplementary file4 (DOCX 598 KB) [file 10548_2023_1019_MOESM4_ESM.docx]

**Supplementary**


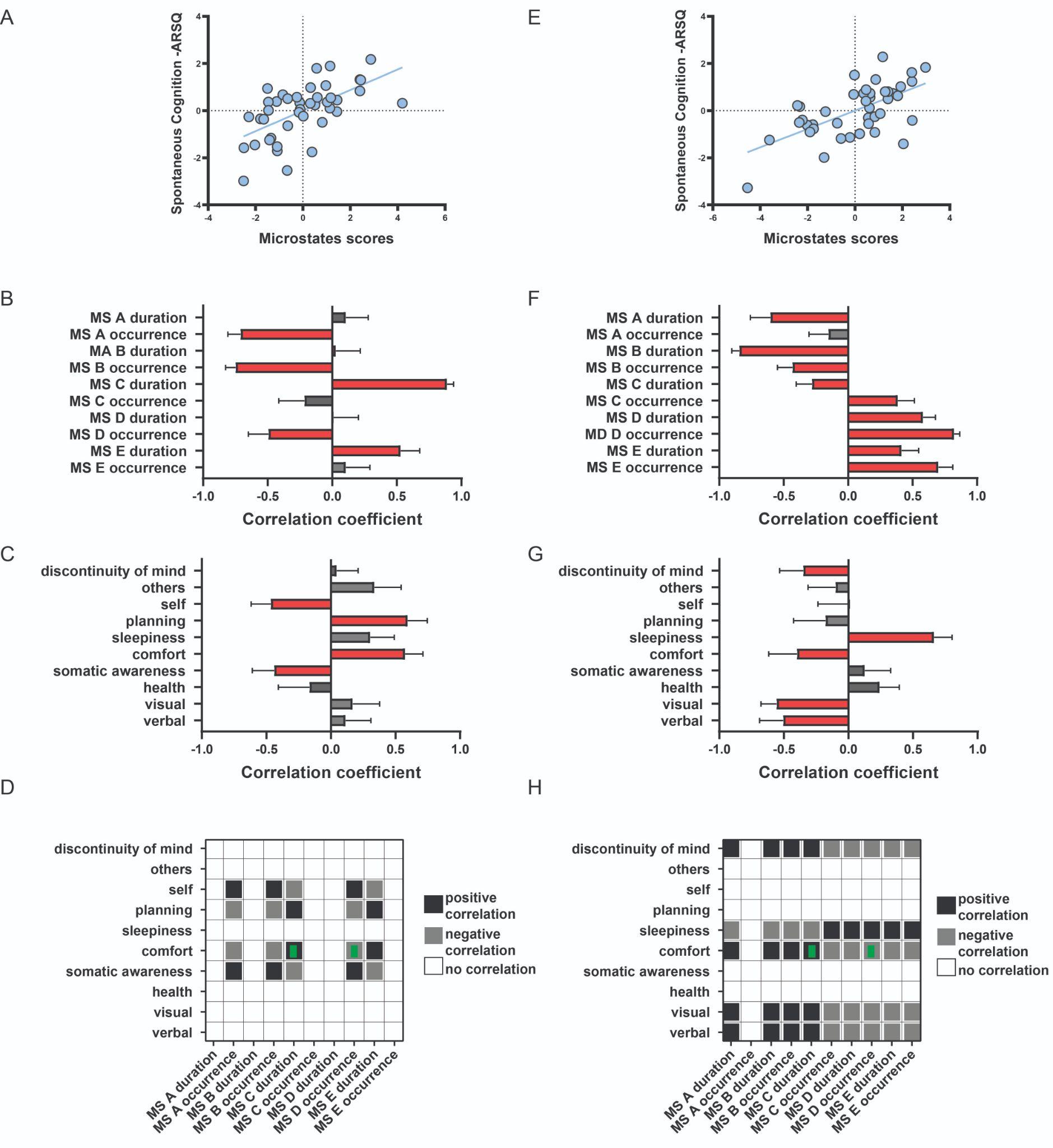


Figure S1. Inter-day association between spontaneous cognition and EEE microstate dynamics. (A) D1 correlation between individual-specific ARSQ scores, microstate duration, and occurrence on day one. (B) D1 correlations between original and composite microstate duration scores. (C) D1 correlations between original and composite ARSQ scores. (D) D1 specific associations between microstate duration and spontaneous thoughts. (E) D2 correlation between individual-specific ARSQ scores and microstate duration and occurrence on day one. (F) D2 correlations between original and composite microstate duration scores. (G) D2 correlations between original and composite ARSQ scores. (H) D2 specific associations between microstate duration and spontaneous thoughts. Errors represent SD. Green color squares mark the consistent D1-D2 associations.

Table S1. Cronbach's alpha measure of internal reliability for personality NEO PI-R and spontaneous thoughts ARSQ questionnaires.

|  | D1 |  | D2 |
| --- | --- | --- | --- |
|  | Cronbach α |  | Cronbach α |
| NEO PI-R |  |  |  |
| N | 0,91 |  | - |
| E | 0,90 |  | - |
| O | 0,87 |  | - |
| A | 0,78 |  | - |
| C | 0,94 |  | - |
|  |  |  |  |
| ARSQ |  |  |  |
| discontinuity | 0,71 |  | 0,79 |
| others | 0,69 |  | 0,68 |
| self | 0,70 |  | 0,72 |
| planning | 0,80 |  | 0,83 |
| sleepiness | 0,76 |  | 0,79 |
| comfort | 0,55 |  | 0,82 |
| somatic awareness | 0,61 |  | 0,72 |
| health | 0,59 |  | 0,66 |
| visual | 0,81 |  | 0,89 |
| verbal | 0,81 |  | 0,62 |
| N1-Anxiety | 0,74 |  | - |
| N2-Hostility | 0,81 |  | - |
| N3-Depression | 0,85 |  | - |
| N4-Self-consciousness | 0,64 |  | - |
| N5-Impulsivness | 0,79 |  | - |
| N6-Vulnerablity to Stress | 0,86 |  | - |
| E1-Warmth | 0,76 |  | - |
| E2-Gregariousness | 0,87 |  | - |
| E3-Assertivness | 0,80 |  | - |
| E4-Activity | 0,83 |  | - |
| E5-Excitement Seeking | 0,53 |  | - |
| E6-Positive Emotions | 0,77 |  | - |
| O1-Fantasy | 0,71 |  | - |
| O2-Aestetics | 0,82 |  | - |
| O3-Feelings | 0,63 |  | - |
| O4-Actions | 0,48 |  | - |
| O5-Ideas | 0,74 |  | - |
| O6-Values | 0,50 |  | - |
| A1-Trust in Others | 0,71 |  | - |
| A2-Straitforwardness | 0,76 |  | - |
| A3-Altruism | 0,67 |  | - |
| A4-Compliance | 0,64 |  | - |
| A5-Modesty | 0,79 |  | - |
| A6-Tendermindedness | 0,56 |  | - |
| C1-Compenteces | 0,71 |  | - |
| C2-Orderliness | 0,73 |  | - |
| C3-Dutifulness | 0,71 |  | - |
| C4-Achievement Striving | 0,85 |  | - |
| C5-Self-discipline | 0,88 |  | - |
| C6-Deliberation | 0,80 |  | - |

D1=day 1, D2=day 2, N=Neuroticism, E=extraversion, O=openness, A=agreeableness, C=conscientiousness.

Table S1. Demographic informaitons os sample size (N), mean and standard deviation (sd) of microstate dynamics, ARSQ and NEO PI-R scores.

|  |  | D1 |  |  |  | D2 |  |  |  | D2-D1 |  |
| --- | --- | --- | --- | --- | --- | --- | --- | --- | --- | --- | --- |
|  | N | mean | sd |  | N | mean | sd |  | N | mean | sd |
| Mean Duration (ms) | |  |  |  |  |  |  |  |  |  |  |
| A | 43 | 69,72360 | 6,72418 |  | 43 | 71,27938 | 8,74198 |  | 43 | 1,55578 | 7,25846 |
| B | 43 | 80,66694 | 13,39760 |  | 43 | 82,51312 | 15,28678 |  | 43 | 1,84618 | 10,65211 |
| C | 43 | 99,07694 | 18,67320 |  | 43 | 96,02892 | 17,31460 |  | 43 | -3,04802 | 16,58838 |
| D | 43 | 71,54000 | 9,50971 |  | 43 | 67,31847 | 14,66965 |  | 43 | -4,22153 | 13,89827 |
| E | 43 | 73,22477 | 9,99484 |  | 43 | 72,95372 | 10,75765 |  | 43 | -0,27104 | 10,95752 |
| Occurrence (Hz) |  |  |  |  |  |  |  |  |  |  |  |
| A | 43 | 1,385082 | 0,486952 |  | 43 | 1,552088 | 0,636239 |  | 43 | 0,167006 | 0,653152 |
| B | 43 | 2,134188 | 0,698935 |  | 43 | 2,336809 | 0,765288 |  | 43 | 0,202620 | 0,666755 |
| C | 43 | 3,048168 | 0,559074 |  | 43 | 2,942664 | 0,655742 |  | 43 | -0,105504 | 0,755577 |
| D | 43 | 1,666927 | 0,820283 |  | 43 | 1,410849 | 0,944884 |  | 43 | -0,256078 | 0,794777 |
| E | 43 | 1,571543 | 0,674025 |  | 43 | 1,567903 | 0,732876 |  | 43 | -0,003640 | 0,703786 |
| ARSQ |  |  |  |  |  |  |  |  |  |  |  |
| discontinuity | 43 | 10,60465 | 2,331355 |  | 43 | 9,76744 | 2,926376 |  | 43 | -0,83721 | 2,707399 |
| others | 43 | 8,74419 | 2,879261 |  | 43 | 8,58140 | 2,985940 |  | 43 | -0,16279 | 3,545153 |
| self | 43 | 10,48837 | 3,073482 |  | 43 | 10,72093 | 3,049790 |  | 43 | 0,23256 | 3,828696 |
| planning | 43 | 9,79070 | 3,681085 |  | 43 | 9,95349 | 3,631565 |  | 43 | 0,16279 | 4,423585 |
| sleepiness | 43 | 7,02326 | 3,097529 |  | 43 | 7,90698 | 3,435210 |  | 43 | 0,88372 | 4,007192 |
| comfort | 43 | 10,90698 | 2,033223 |  | 43 | 10,95349 | 2,716383 |  | 43 | 0,04651 | 2,794156 |
| somatic awareness | 43 | 10,25581 | 3,117485 |  | 43 | 9,16279 | 3,169274 |  | 43 | -1,09302 | 3,293673 |
| health | 43 | 4,83721 | 2,192305 |  | 43 | 4,72093 | 2,174808 |  | 43 | -0,11628 | 3,009582 |
| visual | 43 | 11,74419 | 3,230016 |  | 43 | 11,67442 | 3,530283 |  | 43 | -0,06977 | 3,844572 |
| verbal | 43 | 9,13953 | 3,713434 |  | 43 | 9,20930 | 3,226070 |  | 43 | 0,06977 | 4,278381 |
| NEO PI-R |  |  |  |  |  |  |  |  |  |  |  |
| N1-Anxiety | 37 | 19,43243 | 5,161506 |  | - | - | - |  | - | - | - |
| E1-Warmth | 37 | 19,05405 | 4,594356 |  | - | - | - |  | - | - | - |
| O1-Fantasy | 37 | 22,00000 | 4,778424 |  | - | - | - |  | - | - | - |
| A1-Trust in Others | 37 | 16,91892 | 4,867000 |  | - | - | - |  | - | - | - |
| C1-Compenteces | 37 | 20,43243 | 4,252715 |  | - | - | - |  | - | - | - |
| N2-Hostility | 37 | 14,27027 | 6,039907 |  | - | - | - |  | - | - | - |
| E2-Gregariousness | 37 | 14,51351 | 6,099279 |  | - | - | - |  | - | - | - |
| O2-Aestetics | 37 | 21,21622 | 6,151491 |  | - | - | - |  | - | - | - |
| A2-Straitforwardness | 37 | 20,10811 | 5,541679 |  | - | - | - |  | - | - | - |
| C2-Orderliness | 37 | 18,78378 | 5,175450 |  | - | - | - |  | - | - | - |
| N3-Depression | 37 | 18,62162 | 6,125318 |  | - | - | - |  | - | - | - |
| E3-Assertivness | 37 | 14,18919 | 5,456076 |  | - | - | - |  | - | - | - |
| O3-Feelings | 37 | 23,32432 | 4,177014 |  | - | - | - |  | - | - | - |
| A3-Altruism | 37 | 21,24324 | 3,925737 |  | - | - | - |  | - | - | - |
| C3-Dutifulness | 37 | 22,48649 | 4,388252 |  | - | - | - |  | - | - | - |
| N4-Self-consciousness | 37 | 18,37838 | 4,264526 |  | - | - | - |  | - | - | - |
| E4-Activity | 37 | 17,35135 | 5,308359 |  | - | - | - |  | - | - | - |
| O4-Actions | 37 | 16,48649 | 4,154125 |  | - | - | - |  | - | - | - |
| A4-Compliance | 37 | 17,27027 | 4,787998 |  | - | - | - |  | - | - | - |
| C4-Achievement Striving | 37 | 20,83784 | 5,955919 |  | - | - | - |  | - | - | - |
| N5-Impulsivness | 37 | 17,89189 | 5,496382 |  | - | - | - |  | - | - | - |
| E5-Excitement Seeking | 37 | 17,37838 | 4,296971 |  | - | - | - |  | - | - | - |
| O5-Ideas | 37 | 23,51351 | 5,975048 |  | - | - | - |  | - | - | - |
| A5-Modesty | 37 | 18,45946 | 5,107699 |  | - | - | - |  | - | - | - |
| C5-Self-discipline | 37 | 16,91892 | 6,148073 |  | - | - | - |  | - | - | - |
| N6-Vulnerablity to Stress | 37 | 13,16216 | 5,852414 |  | - | - | - |  | - | - | - |
| E6-Positive Emotions | 37 | 19,18919 | 5,536936 |  | - | - | - |  | - | - | - |
| O6-Values | 37 | 23,24324 | 3,435157 |  | - | - | - |  | - | - | - |
| A6-Tendermindedness | 37 | 21,10811 | 3,133899 |  | - | - | - |  | - | - | - |
| C6-Deliberation | 37 | 15,97297 | 5,469544 |  | - | - | - |  | - | - | - |

D1=day 1, D2=day2

Table S2. Simple bivariate correlations between microstate dynamics and measures of personality NEO PI-R scores.

|  |  |  | D1 |  |  |
| --- | --- | --- | --- | --- | --- |
|  | N | E | O | A | C |
| A mean duration | -0,007 | -0,087 | 0,093 | 0,211 | -0,005 |
| A occurrence | 0,009 | 0,108 | **0,342** | 0,060 | 0,000 |
| B mean duration | -0,160 | -0,135 | -0,124 | -0,042 | 0,023 |
| B occurrence | -0,078 | -0,110 | -0,058 | -0,136 | 0,039 |
| C mean duration | 0,163 | -0,080 | -0,208 | 0,011 | -0,001 |
| C occurrence | 0,054 | 0,119 | -0,070 | -0,077 | 0,135 |
| D mean duration | -0,172 | -0,053 | 0,245 | 0,313 | -0,038 |
| D occurrence | -0,134 | 0,004 | 0,231 | 0,200 | -0,043 |
| E mean duration | -0,021 | 0,032 | -0,036 | 0,038 | -0,065 |
| E occurrence | 0,025 | 0,054 | 0,060 | -0,023 | -0,060 |
|  |  |  |  |  |  |
|  |  |  | D2 |  |  |
|  | N | E | O | A | C |
| A mean duration | 0,084 | 0,119 | -0,190 | 0,033 | 0,055 |
| A occurrence | -0,202 | 0,166 | -0,104 | -0,035 | 0,221 |
| B mean duration | -0,084 | 0,027 | 0,040 | -0,032 | -0,046 |
| B occurrence | -0,014 | -0,095 | 0,247 | -0,081 | -0,129 |
| C mean duration | 0,235 | -0,200 | -0,151 | 0,116 | -0,130 |
| C occurrence | 0,033 | -0,152 | -0,028 | 0,141 | -0,108 |
| D mean duration | -0,011 | -0,158 | -0,141 | -0,096 | 0,001 |
| D occurrence | 0,014 | -0,071 | 0,039 | 0,010 | -0,026 |
| E mean duration | 0,072 | 0,184 | -0,067 | -0,282 | 0,168 |
| E occurrence | 0,008 | 0,175 | -0,099 | -0,112 | 0,265 |

D1=day 1, D2=day 2, D2-D1=difference scores. N=Neuroticism, E=extraversion, O=openness, A=agreeableness, C=conscientiousness. Bold values represent p<0,05.

Table S3. Simple bivariate correlations between microstate dynamics and measures of personality NEO PI-R scores.

|  |  |  |  |  | D1 |  |  |  |  |  |
| --- | --- | --- | --- | --- | --- | --- | --- | --- | --- | --- |
|  |  |  |  |  |  |  | somatic awareness |  |  |  |
|  | discontinuity | others | self | planning | sleepiness | comfort |  | health | visual | verbal |
| A mean duration | 0,130 | -0,129 | 0,136 | 0,052 | 0,059 | 0,220 | -0,040 | -0,138 | 0,208 | 0,303 |
| A occurrence | -0,006 | -0,147 | 0,246 | -0,179 | -0,036 | -0,034 | 0,323 | -0,004 | 0,206 | 0,058 |
| B mean duration | **0,325** | -0,113 | -0,034 | -0,085 | -0,078 | -0,175 | -0,216 | 0,053 | 0,023 | -0,033 |
| B occurrence | 0,229 | -0,130 | 0,125 | -0,229 | -0,196 | **-0,454** | 0,046 | 0,205 | -0,018 | -0,111 |
| C mean duration | 0,011 | 0,177 | **-0,334** | 0,224 | 0,128 | 0,236 | -0,291 | -0,199 | 0,062 | 0,019 |
| C occurrence | -0,310 | 0,061 | -0,078 | -0,019 | -0,012 | -0,125 | 0,122 | 0,091 | -0,123 | 0,006 |
| D mean duration | 0,034 | 0,086 | 0,297 | -0,004 | -0,005 | 0,149 | 0,183 | -0,070 | -0,022 | 0,169 |
| D occurrence | -0,126 | 0,099 | 0,306 | -0,124 | -0,067 | -0,078 | 0,244 | -0,048 | -0,011 | -0,034 |
| E mean duration | 0,143 | -0,105 | -0,042 | 0,154 | 0,239 | **0,435** | 0,057 | 0,085 | -0,017 | 0,036 |
| E occurrence | -0,037 | 0,005 | 0,134 | 0,108 | 0,099 | **0,327** | 0,134 | 0,121 | 0,074 | 0,005 |
|  |  |  |  |  |  |  |  |  |  |  |
|  |  |  |  |  | D2 |  |  |  |  |  |
|  |  |  |  |  |  |  | somatic awareness |  |  |  |
|  | discontinuity | others | self | planning | sleepiness | comfort |  | health | visual | verbal |
| A mean duration | 0,217 | -0,023 | -0,323 | 0,294 | -0,307 | 0,003 | 0,012 | -0,074 | 0,025 | 0,308 |
| A occurrence | 0,105 | 0,028 | -0,150 | **0,394** | 0,034 | 0,063 | -0,057 | 0,090 | -0,164 | 0,160 |
| B mean duration | 0,110 | 0,005 | -0,148 | 0,186 | **-0,442** | -0,043 | 0,062 | -0,083 | 0,195 | 0,103 |
| B occurrence | -0,053 | 0,064 | 0,028 | 0,125 | -0,247 | 0,050 | 0,036 | 0,192 | 0,151 | 0,129 |
| C mean duration | 0,048 | 0,076 | -0,085 | -0,085 | -0,075 | 0,077 | -0,015 | -0,272 | 0,166 | 0,141 |
| C occurrence | -0,083 | -0,076 | -0,032 | **-0,353** | 0,092 | 0,047 | -0,310 | -0,301 | -0,175 | -0,009 |
| D mean duration | -0,117 | -0,055 | 0,028 | -0,236 | 0,226 | -0,080 | -0,007 | -0,039 | -0,137 | 0,044 |
| D occurrence | -0,217 | -0,032 | 0,196 | -0,186 | **0,327** | -0,047 | 0,027 | 0,231 | -0,216 | -0,101 |
| E mean duration | 0,028 | 0,005 | 0,106 | -0,037 | 0,136 | -0,143 | 0,089 | -0,010 | 0,055 | -0,209 |
| E occurrence | 0,009 | 0,100 | 0,233 | 0,053 | **0,406** | -0,080 | 0,145 | 0,155 | -0,059 | -0,197 |
|  |  |  |  |  |  |  |  |  |  |  |
|  |  |  |  |  | D2-D1 |  |  |  |  |  |
|  |  |  |  |  |  |  | somatic awareness |  |  |  |
|  | discontinuity | others | self | planning | sleepiness | comfort |  | health | visual | verbal |
| A mean duration | -0,095 | -0,184 | **0,328** | -0,064 | -0,146 | -0,250 | 0,155 | -0,148 | **-0,351** | -0,255 |
| A occurrence | -0,171 | -0,239 | -0,019 | -0,133 | -0,149 | **-0,334** | -0,104 | -0,182 | -0,296 | 0,010 |
| B mean duration | 0,093 | -0,106 | 0,132 | -0,064 | -0,027 | -0,222 | 0,018 | -0,305 | -0,115 | **-0,335** |
| B occurrence | 0,143 | -0,168 | -0,031 | -0,210 | -0,132 | -0,244 | -0,191 | -0,319 | -0,024 | -0,198 |
| C mean duration | -0,275 | -0,033 | 0,063 | -0,129 | 0,227 | 0,181 | 0,310 | 0,115 | 0,178 | -0,086 |
| C occurrence | -0,139 | 0,248 | 0,115 | -0,148 | 0,179 | 0,119 | 0,013 | 0,090 | 0,262 | 0,072 |
| D mean duration | 0,132 | -0,071 | 0,209 | -0,132 | 0,020 | 0,031 | 0,143 | -0,052 | -0,077 | 0,160 |
| D occurrence | **0,334** | 0,064 | 0,061 | 0,105 | 0,015 | 0,069 | -0,125 | 0,110 | 0,040 | 0,125 |
| E mean duration | 0,155 | -0,010 | 0,130 | 0,076 | -0,139 | 0,007 | 0,095 | 0,060 | -0,155 | 0,098 |
| E occurrence | 0,021 | 0,034 | -0,059 | 0,188 | -0,056 | 0,116 | -0,173 | 0,233 | -0,027 | 0,240 |

D1=day 1, D2=day 2, D2-D1=difference scores. Bold values represent p<0, 05.


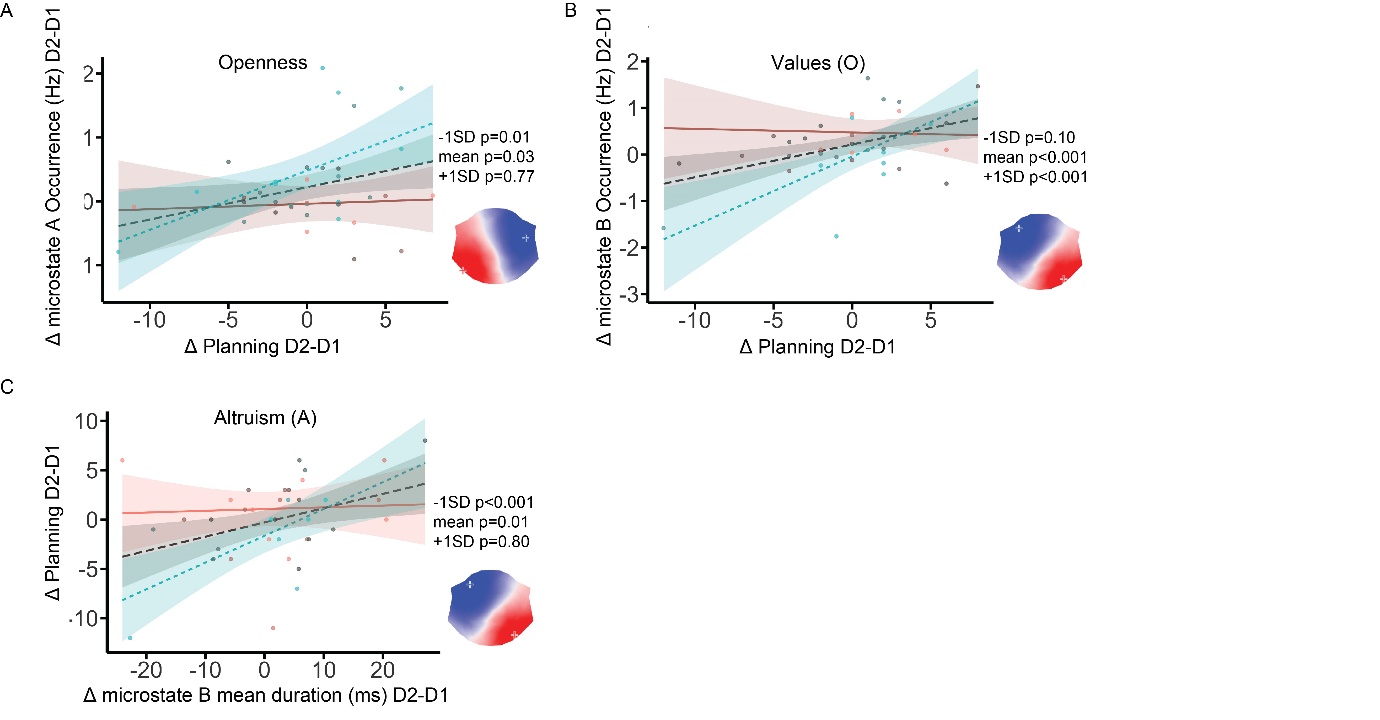


Figure S2. Personality moderates inter-day associated changes in spontaneous thoughts and microstate occurrence. (A) Openness personality traits moderate the association between inter-day changes in microstate A occurrence and Planning. (B) Altruism personality traits moderate the association between intr-day changes in Planning and microstate B mean duration. (C) Values personality traits moderate the association between inter-day changes in microstates B occurrence and Planning. Blue dots and regression lines represent individuals with one standard deviation below the mean (-1SD) on personality trait distribution. Red dots and regression lines represent individuals with one standard deviation above the mean (+1SD) on personality traits.

Table S5. Moderation analysis results for A, B microstates & *Planning*.

| *Slope* | *Predictors* |  | *Estimates* | *CI* | | *t* | *p* |
| --- | --- | --- | --- | --- | --- | --- | --- |
| Planning | Openness × MS A occurrence |  | -0.52 | -1.04 | -0.01 | -2.01 | **0.052** |
| -1SD | low Openness |  | 5.34 | 1.84 | 8.85 | 3.1 | **0.01** |
| mean | mean Openness |  | 2.82 | 0.99 | 4.66 | 3.13 | **0.03** |
| +1SD | high Openness |  | 0.3 | -2.43 | 3.03 | 0.23 | 0.77 |
| Planning | Altruism × MS B duration |  | -0.03 | -0.05 | -0.01 | -2.59 | **0.014** |
| -1SD | low Altruism |  | 0.27 | 0.12 | 0.43 | 3.54 | **0.00** |
| mean | mean Altruism |  | 0.14 | 0.03 | 0.26 | 2.65 | **0.01** |
| +1SD | high Altruism |  | 0.02 | -0.12 | 0.16 | 0.25 | 0.80 |
| MS B | Values × planning |  | -0.02 | -0.04 | 0.00 | -2.27 | **0.03** |
| -1SD | low Values |  | 0.15 | 0.06 | 0.23 | 3.49 | **0.10** |
| mean | mean Values |  | 0.07 | 0.02 | 0.12 | 3.13 | **0.00** |
| +1SD | high Values |  | -0.01 | -0.09 | 0.07 | -0.19 | **0.00** |


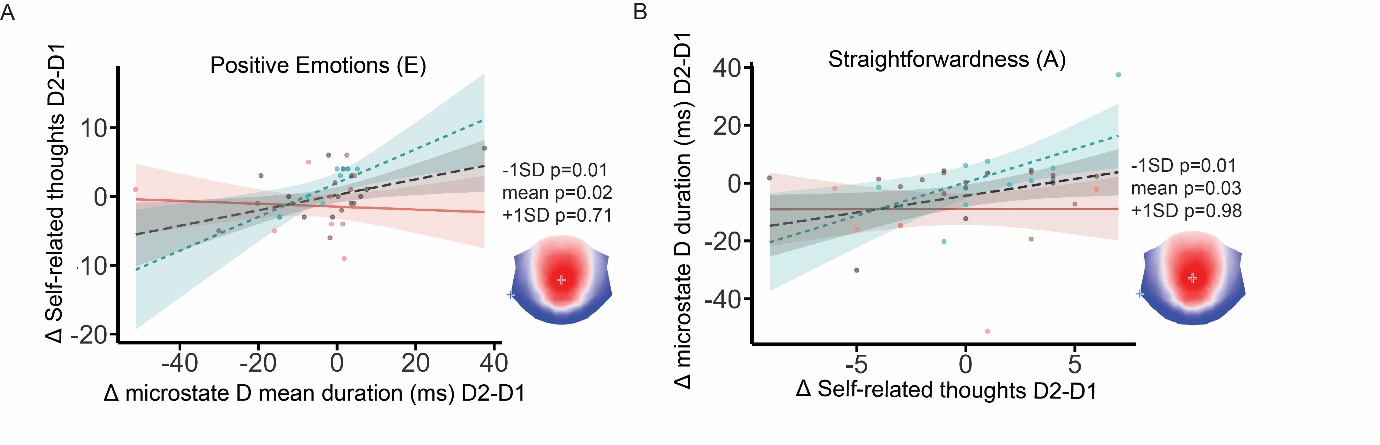


Figure S3. Personality moderates inter-day associated changes in spontaneous thoughts and microstate occurrence. (A) Positive Emotions and personality traits moderate the association between inter-day changes in Self-related thoughts and microstate D mean duration. (F) Straightforwardness personality traits moderate the association between inter-day changes in microstates D mean duration and Self-related thoughts. Blue dots and regression lines represent individuals with one standard deviation below the mean (-1SD) on personality trait distribution. Red dots and regression lines represent individuals with one standard deviation above the mean (+1SD) on personality traits.

Table S5. Moderation analysis results for D microstate & *Self*.

| *Slope* | *Predictors* | *Estimates* | *CI* | | *t* | *p* |
| --- | --- | --- | --- | --- | --- | --- |
| Self | Positive Emotion × MS D duration | -0.02 | -0.04 | -0.00 | -2.43 | **0.021** |
| -1SD | low PosEmo | 0.25 | 0.08 | 0.42 | 2.93 | **0.01** |
| mean | mean PosEmo | 0.11 | 0.02 | 0.2 | 2.51 | **0.02** |
| +1SD | high PosEmo | -0.02 | -0.13 | 0.09 | -0.38 | 0.71 |
| MS D | Straightforwardness × Self | -0.29 | -0.55 | -0.03 | -2.27 | **0.03** |
| -1SD | low StraiF | 2.31 | 0.69 | 3.92 | 2.91 | **0.01** |
| mean | mean StraiF | 1.16 | 0.1 | 2.22 | 2.23 | **0.03** |
| +1SD | high StraiF | 0.01 | -1.31 | 1.34 | 0.02 | 0.98 |
